# Assessing food availability and healthier options in an urban Chinese university: a case study using the Chinese Nutrition Environment Measurement Survey for Stores (C-NEMS-S)

**DOI:** 10.1186/s12889-023-17415-8

**Published:** 2024-01-02

**Authors:** Xingbo Li, Haiyue Wang, Hendra Manafe, Andrea Braakhuis, Zengning Li, Rajshri Roy

**Affiliations:** 1 Nutrition and Dietetics, Faculty of Medical and Health Sciences, The University of Auckland, Auckland, 1010 New Zealand; 2 The First Hospital of Hebei Medical University, Shijiazhuang, Hebei 050031 China; 3 Hebei Province Key Laboratory of Nutrition and Health SZX2021021, Shijiazhuang, Hebei 050031 China; 4 Charles Perkins Centre, University of Sydney, Sydney, 2006 Australia; 5 Nutrition and Dietetics, Sydney School of Nursing, Faculty of Medicine and Health, The University of Sydney, Sydney, 2006 Australia

**Keywords:** Food environment, University, Food availability, Healthier options, Price

## Abstract

Young adults (18–24 years) in universities are frequently exposed to an environment that promotes unhealthy eating behaviors. Using a validated tool, the Chinese Nutrition Environment Measurement Survey for Stores (C-NEMS-S), we assess the food availability and healthier options in a large, urban Chinese university. We employed C-NEMS-S for scoring criteria and weighting. A total of 52 on-campus canteen outlets were audited in an urban university located in Shijiazhuang City, China. General food outlets (*n* 43) and self-served food outlets (*n* 7) were further categorized into eight subtypes. Beverage outlets (*n* 2) were discussed separately from food outlets. C-NEMS-S scores were significantly different across food outlet types (*P* = 0.0024), especially between noodle and rice outlets (*P* = 0.0415). Food availability scores for starchy tubers (*P* < 0.001), dry beans (*P* < 0.001), vegetables (*P* = 0.0225), and fruits (*P* < 0.001) were significantly different across food outlet subtypes. Healthier options were scarce and only appeared in “grains” (*n* 2) and “meat and poultry” (*n* 2) categories. Further research on improving the accustomed audit tool and assessing university student diet quality is warranted.

**Supplementary Information:**

The online version contains supplementary material available at 10.1186/s12889-023-17415-8.

## Introduction

In 2016, more than 1.9 billion adults were overweight globally, where 650 million were considered obese [1]. Obesity doubled in over 70 countries from 1980 to 2015, leading to four million deaths related to a high BMI [2]. Obesity is caused by multiple factors, largely centered on an energy imbalance. Maintaining a balanced, healthy diet and lifestyle is crucial for reducing the risk of and managing obesity, with global guidelines recommending a low-energy diet and physical activity [3, 4]. Despite widespread education and healthy eating campaigns, the prevalence of excessive body weight remains stubbornly high in many countries such as the United States, the United Kingdom, and Mexico while steadily climbing in others, including India and China [5].

The prevalence of overweight and obesity in China among adults, children aged 6–17 years, and children below 6 years were 50.7%, 19.0%, and 10.4%, respectively [6]. Young adulthood (18–24 years) is a formative life stage for understanding how the obesity rate dramatically increased from adolescence to adults. Young adults make up much of the tertiary education sector age group and are particularly at high risk of developing unhealthy dietary habits and disordered eating patterns [7], which could subsequently lead to health problems such as obesity and diabetes [7, 8]. Firstly, transitioning from high school to university can be a challenging time that requires a lot of adjustment [9]. Food guidelines worldwide promote variety, while limiting added sugar, saturated fat, and salt [10]. Students may experience high levels of stress, poor time management, and a lack of cooking skills, leading to an increased reliance on convenience foods that are often high in calories, sugar, and fat [11–15]. Additionally, young adults attending university may lack knowledge about proper nutrition and healthy eating habits. Many students have never been responsible for planning and preparing their own meals before and may not know how to choose and prepare healthy foods [16–18]. In the meantime, students may feel the pressure to conform to unhealthy eating habits, such as binge drinking or consuming large amounts of unhealthy food, to fit in with their peers [19–21].

Young adults in universities are frequently exposed to an environment that promotes unhealthy eating behaviors. The impact from university food environment originates from its components and is then amplified with perceptions of students about the food environment: taste, price, and accessibility [22]. For example, university cafeterias often offer a wide range of unhealthy foods and drinks, and vending machines stocked with unhealthy snacks and sugary beverages are usually readily available. Additionally, university social events often involve unhealthy foods and alcoholic drinks, which can contribute to poor dietary behavior [22, 23]. The gap between students’ understanding of what “healthy food” is and the objective assessment outcomes of its healthiness may have disguised underlying needs to modify the university food environment [24].

University students have reported the impact of the obesogenic food environment on their dietary behaviours and suggested that improved food environment would mitigate this issue [25]. Unlike Western universities, Chinese universities manage canteens directly and focus on providing affordable and convenient eating options thanks to government subsidies for university dining practices. Existing Chinese university food environment studies focus on food waste [26], food safety [27], and food hygiene [28]. In contrast, little research has been done on the link between the university food environment, the nutritional value of food on offer, and the subsequent dietary behaviors of Chinese students. Despite numerous interventional studies being conducted, questions remain as to what the food environment is, in universities in China. The characterization of the Chinese university food environment deserves attention [29].

Before considering dietary intake or food environment interventions, the Chinese university food environment must be systematically studied to inform further research in such settings. A validated tool, the Chinese Nutrition Environment Measurement Survey for Stores (C-NEMS-S), was developed specifically for China’s distinct food service patterns and hence, was used to audit the university food environment [30]. This study aims to establish the context of Chinese university campus food environment from three aspects: food availability, healthier food options, and additional features.

## Materials and methods

### Research design and setting

All food outlets assessed in this investigation were located within three canteens in an urban university in Shijiazhuang City, China and was conducted between February and March 2022. The university had two campuses and held more than 19,000 full-time students. The audit was cross-sectional and involved 52 outlets located in the canteens. This particular university was chosen for this audit because its food environment setting is similar to those found in other typical Chinese universities. However, this study is pioneer research regarding university food environments in China and we have no intention to generalize any of our results on a national or international level at this point. Since this study did not involve any animal or human interventions, ethical approval was not required.

### Food outlet selection

The authors collected information from food outlets through on-site visits to the university canteens. The inclusion criteria were as follows, (1) actively operating food outlets located within the university canteen and, (2) the ability to serve hot food. The maximum operational capacity of the audited food environment was 70 outlets; 11 outlets were excluded because they were vacant. Certain outlets occupied two or more outlet spaces but were considered one outlet since the foods and beverages served were the same across their occupancy. Among the 52 operating outlets that were ultimately audited, 7 were self-served outlets, and 2 were beverage outlets. Features of each type of outlet category were explored in subsequent analysis.

### Food environment audit tool

One of the most widely used tools to measure food environments is the Nutrition Environment Measurement Survey (NEMS). Based on NEMS, Glanz, Sallis [31] observed consumer and community food environment that involved stores selling food and beverage to customers. Their adapted tool featured reliable measurement of food environment components with closer proximity and higher availability, which resembled the food environment in university settings. Chinese food environment, especially within university campus settings, differ from the Western food environment and so we employed a validated Chinese Version of the Nutrition Environment Measurement Tool for Stores (C-NEMS-S) for this study [30]. The advantage of using the C-NEMS-S in Chinese university settings included adjustments to food categories that reflect the actual food composition, to definitions of foods that belong to each category, and scoring criteria and weighting. The research team dropped components from the original C-NEMS-S that were considered not applicable when piloted in the current setting.

### Audit tool pilot testing

The C-NEMS audit tool was pilot tested for the current research setting in February 2022. Four canteen outlets in Zhongshan campus were randomly selected and audited using the original C-NEMS-S. The results of pilot test indicated that five categories were not applicable in the university canteen food environment, including: dietary oils, milk, bread, instant noodles, and beverages. Thus, these five categories were removed from subsequent audit, as shown in Table 1. The investigator was accompanied by each of the three canteen managers during the pilot audit because this was necessary to achieve cooperation from canteen outlet staff. After pilot audit at each canteen, the investigator interviewed the managers on their general opinion about the university food environment and specifics on their canteens. The information was recorded and presented in the Results section where relevant.

**Table 1:** Overview of the scoring criteria that were applicable or not applicable in the C-NEMS-S tool used in this audit

Outlet scoring criteria	Original scores available for each outlet	Actual scores available for each outlet
Dietary Oils	0 to 6	Criteria Removed
Milk	0 to 5	Criteria Removed
Bread	0 to 5	Criteria Removed
Instant Noodles	0 to 5	Criteria Removed
Beverages	0 to 18	Criteria Removed
Availability	0 to 48	0 to 19
Price	-14 to 26	-2 to 4
Quality^a^	0 to 9	0 to 9
Total Score Range	-14 to 83	-2 to 32

^a^Unlike the original C-NEMS-S, we assigned a universal quality score of 3 by default for applicable food categories served at each outlet

**Fig. 1:**
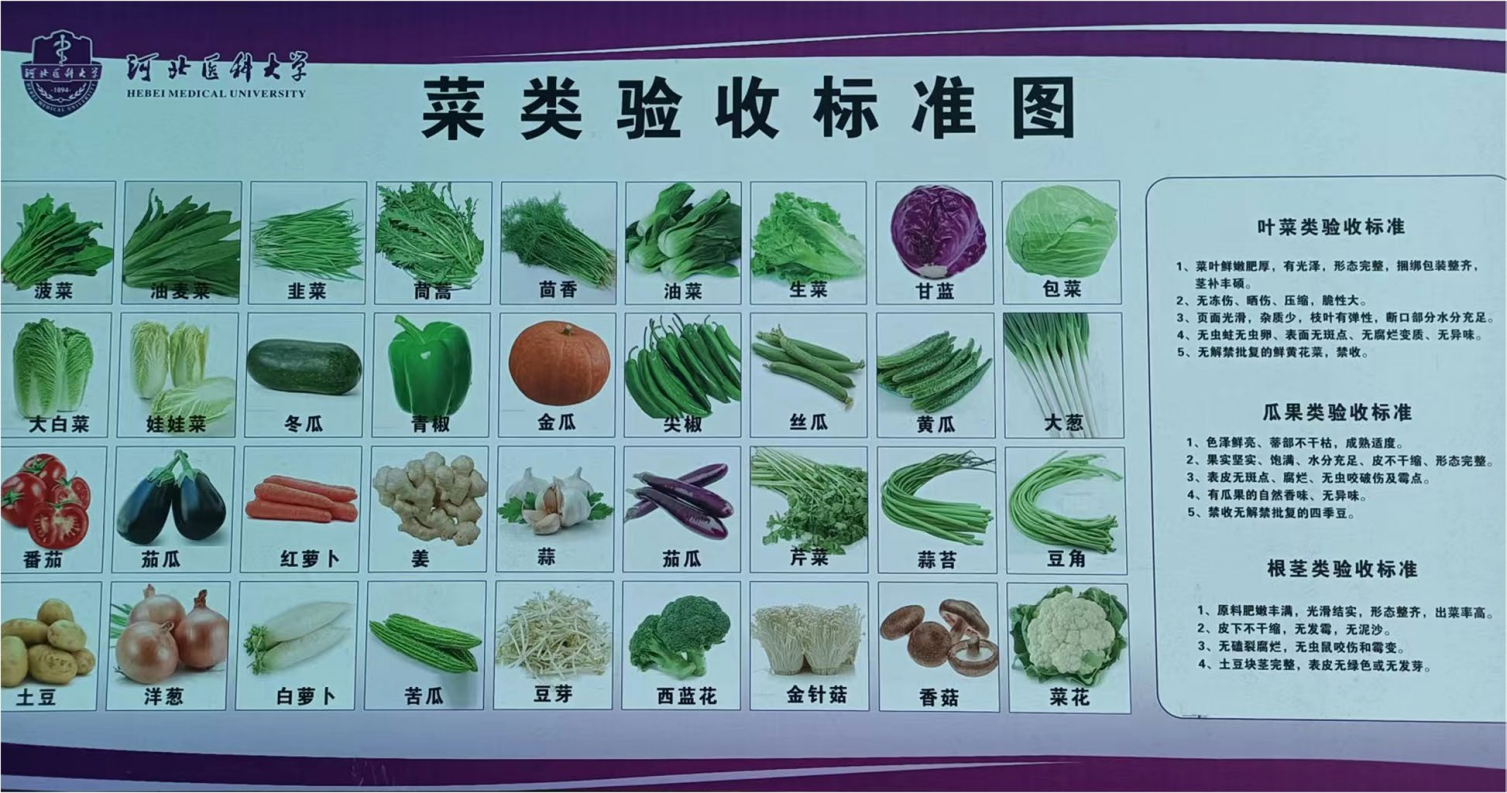
Food ingredient quality checklist with graphic illustrations placed at university canteen docking bays

C-NMES-S scores vegetables, fruits, and seafood based on the quality of food ingredients based on their freshness. The university canteen in this study implements mandatory freshness check at food ingredient purchase delivery every day. A standardized chart for acceptable food ingredient freshness was displayed at the docking bay for quality control staff to refer to, as shown in Fig. 1. The author conducted an independent quality check on two non-consecutive weekdays. The freshness of food ingredients assessed by university canteen staff and independently by the researcher reached an agreement. Hence, the freshness scored for vegetables sold in university canteen food outlets were given the highest scores on the C-NEMS-S audit tool except for pickled vegetables. Pickled vegetables counted towards the vegetable criteria but had one score deducted from the freshness score because they were preserved.

### Food outlet categories

Based on cultural norms of the Chinese diet traditions and expert consultation, we developed categories for the food outlets to accommodate for the heterogeneity across different food outlet categories. Using these categories, we were able to compare C-NEMS-S scores within or across groups. As shown in Table 2, all food outlets were categorized into general food outlets, self-served outlets, or beverage outlets. General food outlets and self-served outlets were further categorized according to what was served for subsequent statistical analysis. Beverage outlets were excluded from such analysis and discussed in a separate section later. An example photo of each outlet subtype has been included. The photos generally reflected what the students would see when they approach the outlet. Some outlets displayed food and beverage that are ready to serve (for example, creperie) while others only displayed a menu for students to choose from (for example, burgers).

**Table 2:**
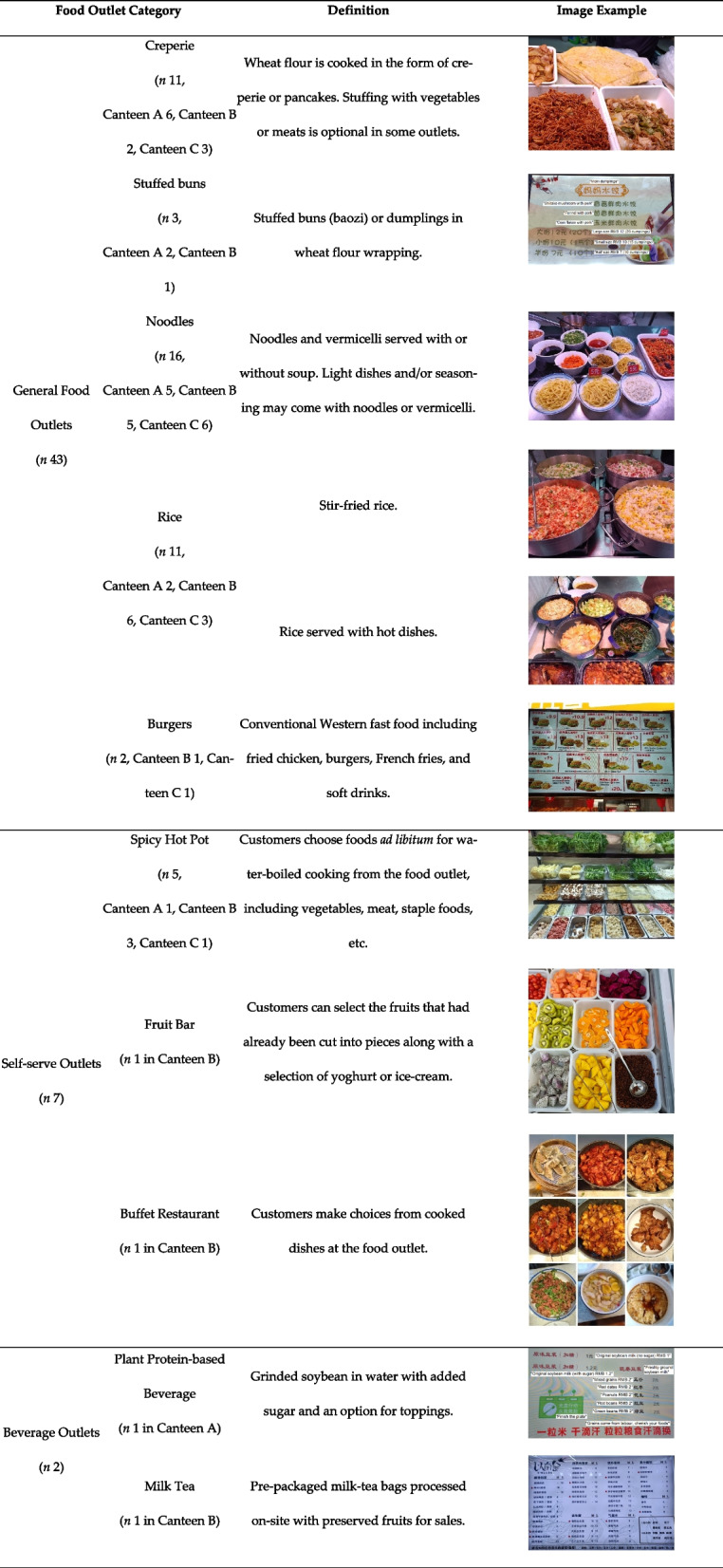
Food categories developed in a Chinese urban university. The number of outlets in each subtype in Canteens A, B, and C have been specified, respectively

### Audits

One investigator conducted three independent audits of all food outlets on a university campus during weekdays from 10:00 am to 11:30 am. The outlets were classified into three main categories (canteen, self-served, and beverage) and further divided into 10 sub-categories. Each outlet was labeled using a combination of a capital letter indicating the major category and two Arabic numbers. For example, the 11th canteen outlet would be labeled C11. The three investigators’ professional backgrounds were from human nutrition, biotechnology, and clinical pediatrics, respectively. They were trained for food environment audits by using C-NEMS-S on outlets C01, C02, and C03. Where disagreements were found, the investigators discussed on how the tool should be interpreted until a consensus could be reached. During the scoring phase, three investigators independently evaluated each outlet’s availability of food, healthier options, price, and quality. In case of any disagreements, the investigators consulted an expert to reach a consensus, which were documented.

### Statistical analysis

Data were processed in GraphPad Prism for Windows (version 10.0.3, Boston, serial number GPS-1121218-L###-#####) and all data analysis were performed with built-in statistical packages. Graphs were generated to represent the relative ranking of scores within each food outlet category and composite C-NEMS-S scores. The median and interquartile range (IQR) were computed within each outlet type, and box plots were generated for composite scores. We divided the actual scores of each food category by the maximum available scores in its corresponding category to yield a percentage value that is subsequently used for statistical analysis. The total scores and food availability scores among different food outlet types were analyzed using one-way ANOVA. Inter-rater reliability was analyzed using Fleiss’s kappa among three investigators [32]. Statistical significance was found when *P* < 0.05 for this study.

## Results

The audit covered 52 food outlets across two urban campuses in a university and 3 food court food service environments. This section summarized the findings from our university food environment audit using C-NEMS-S tool. It began with an overview of the C-NEMS-S scores of the audited university’s on-campus food environment. Next, the food availability scores were further analysed based on different food outlet types. Then, audit outcomes regarding healthier food options and their prices were synthesized. Lastly, the inter-rater reliability results were described.

### Overview of the university food environment

Three canteens were located within the campus where the food environment audit was conducted, namely Canteens A, B, and C. Two of the canteens were adjacent to the student apartment while the other canteen was located at the basement level of the main teaching building, as shown in Table 3. The proximity of canteens to student activities facilitated student access to foods and beverages in university canteens. The opening hours primarily covered the conventional hours of breakfast, lunch, and dinner, respectively. Out of the 50 outlets audited, 11 scored 5 or less out of 32 available scores. Two self-served outlets scored 16, which was the highest score achieved during the audit.

**Table 3:** Summary table of descriptive features of the three canteens in the audited university food environment

Canteen	No. of Outlets	Outlet Types	Spatial Arrangement	Entrances	Opening Hours	Outlet Menus	Proximity
Canteen A	16^a^	6	Ground Floor	East	06:00–09:00 Breakfast 11:00–13:00 Lunch 17:00–20:00 Dinner	Developed by direct employees of the university canteen.	Student Apartment
Canteen B	21^a^	9	First Floor	East		Third-party franchisees managed by the university.	Student Apartment
Canteen C	15	6	Basement Level 1	South and North		Canteen outsourced to a commercial group.	Main Teaching Building
Total	52						

^a^One beverage outlet was present in each of these two canteens. The beverage outlets were excluded from statistical analysis

Two beverage outlets were audited in the study: one selling plant protein-based hot beverages (PPB) and the other selling milk tea. The PPB outlet was located in Canteen A, serving drinks made from dry soybeans with added sucrose while offering additional toppings options including grinded mix grains, red dates, peanuts, red beans, or mung beans at an extra cost.

The milk tea outlet was located independently next to the self-served spicy hot pot outlet in Canteen B. The fruit tea used either canned or pre-packaged fruits with seasoning instead of freshly prepared fruits. Although it was categorized as milk tea, the final drinks contained no dairy products but rather seasoned with non-dairy creamers. Customers could customize their drinks by choosing the amount of added sugar (full, 70%, half, or 30%) and the temperature (iced, reduced ice, no ice, room temperature, warm, hot, or very hot).

**Fig. 2:**
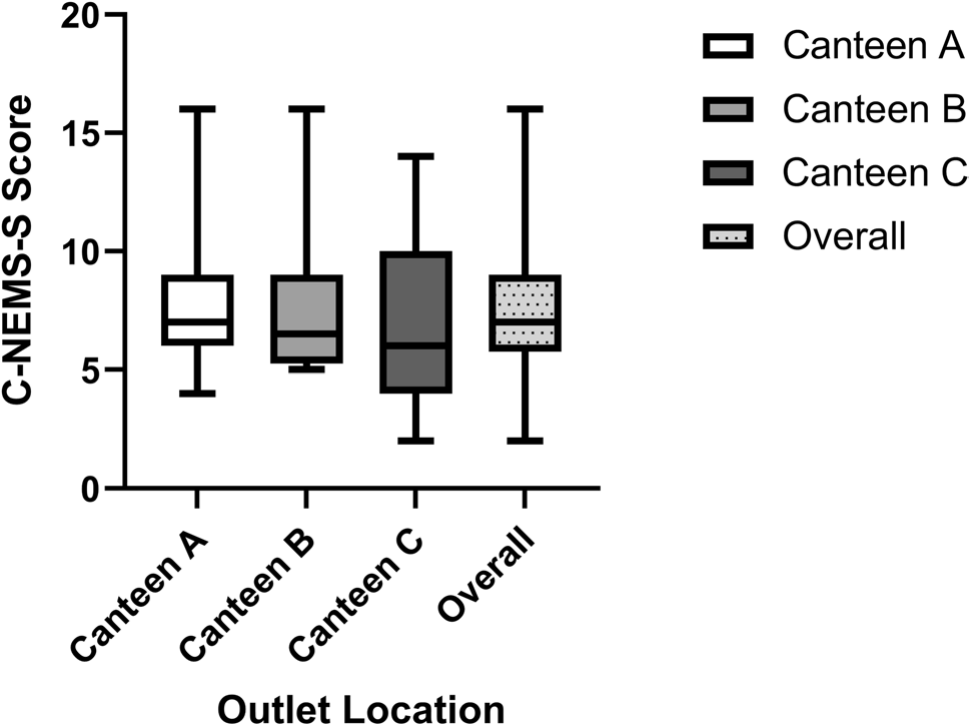
Total C-NEMS-S scores in Canteens A, B, and C. The overall total C-NEMS-S score was plotted as a comparison. The boxes showed the score range from 1st to 3rd quartiles for each canteen with the median marked with a line in the box. The top and bottom bars outside each box showed the maximum and minimum values found in that canteen, respectively

The total score differences among three canteens were not statistically significant. As illustrated in Table 4, comparison of the total C-NEMS-S scores found that the 1st quartile and median total scores of Canteen C (4.00, 6.00) were lower than Canteen A (6.00, 7.00) and Canteen B (5.25, 6.50). However, the 3rd quartile of Canteen C (10.00) was higher than Canteen A (9.00) and Canteen B (9.00), suggesting that the distribution of total scores of outlets in Canteen C was slightly skewed towards both ends. In Canteen B, the outlets clustered near its median and 1st quartile, as shown in Fig. 2.

**Table 4:** Total scores and the percentile and data range for canteens A, B, and C

	Canteen A	Canteen B	Canteen C	Overall
No. of outlets	15	20	15	50
Minimum	4.00	5.00	2.00	2.00
1st Quartile	6.00	5.25	4.00	5.75
Median	7.00	6.50	6.00	7.00
3rd Quartile	9.00	9.00	10.00	9.00
Maximum	16.00	16.00	14.00	16.00
Range	12.00	11.00	12.00	14.00

**Fig. 3:**
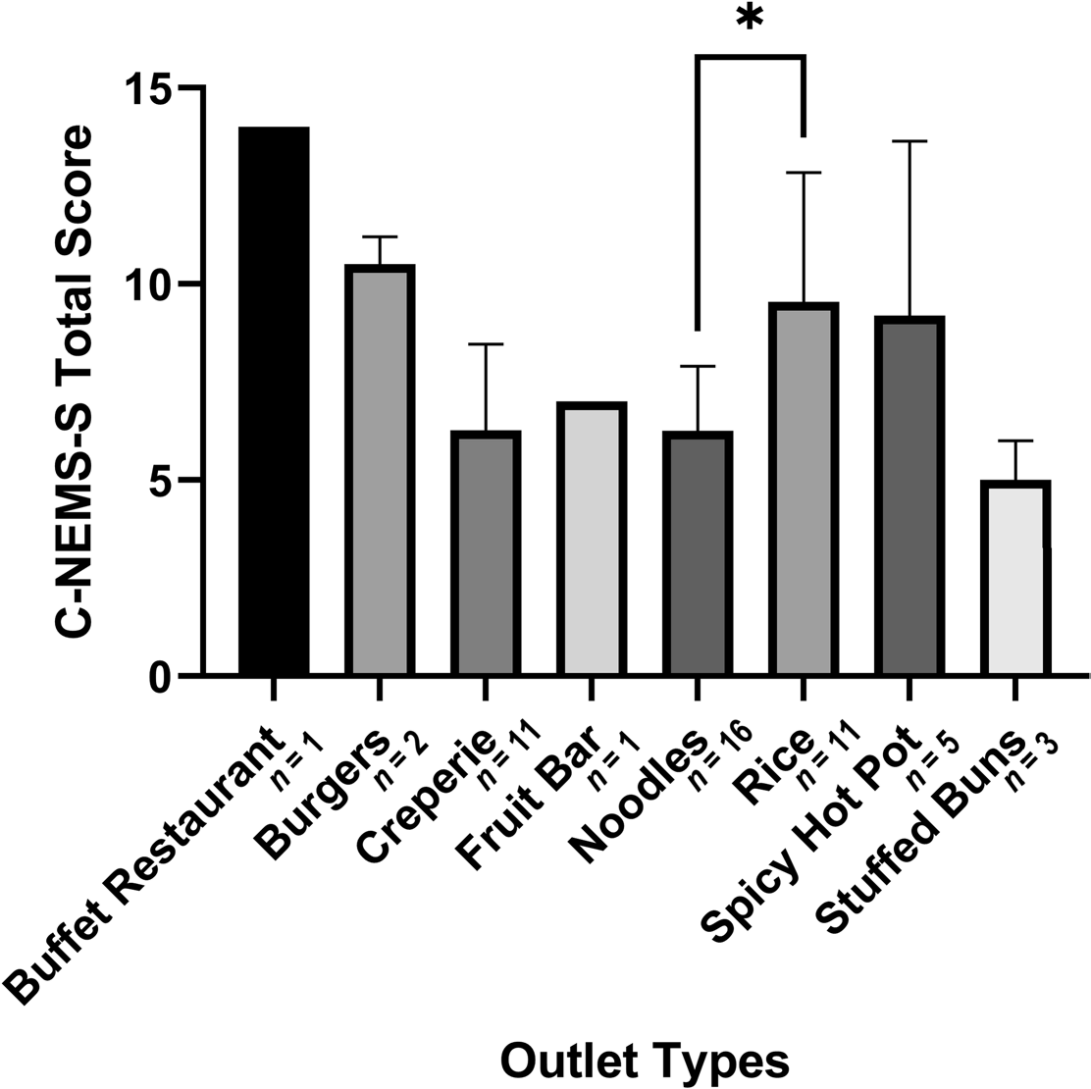
Average C-NEMS-S total score among different sub-categories of food outlets. Pairwise comparison with statistical significance was marked on the graph with an asterisk. Error bars represented the standard error of the scores in each outlet type. Buffet restaurant and fruit bar had no error bars because there was only 1 outlet in each type, respectively. The number of outlets found in each type has been labelled behind the subtype names on the horizontal axis

One-way ANOVA showed that C-NEMS-S scores were significantly different across food outlet types (*P* = 0.0024) where the difference between the total scores of noodle outlets and rice outlets were the most prominent (*P* = 0.0415). The large number of noodle (*n* 16) and rice (*n* 11) outlets combined with the differences in their total scores could have contributed to such results. Pairwise comparison across other outlet types resulted in no statistical significance, as illustrated in Fig. 3.

### Food availability scores

In this section, we first compared the availability scores of each food category in outlets of each subtype. Multiple one-way ANOVA analysis suggested that the scores for starchy tubers (*P* < 0.001), dry beans (*P* < 0.001), vegetables (*P* = 0.0225), and fruits (*P* < 0.001) were significantly different across outlet subtypes. Such findings were consistent with the distinct collection of dishes cooked at each subtype of food outlets.

**Fig. 4:**
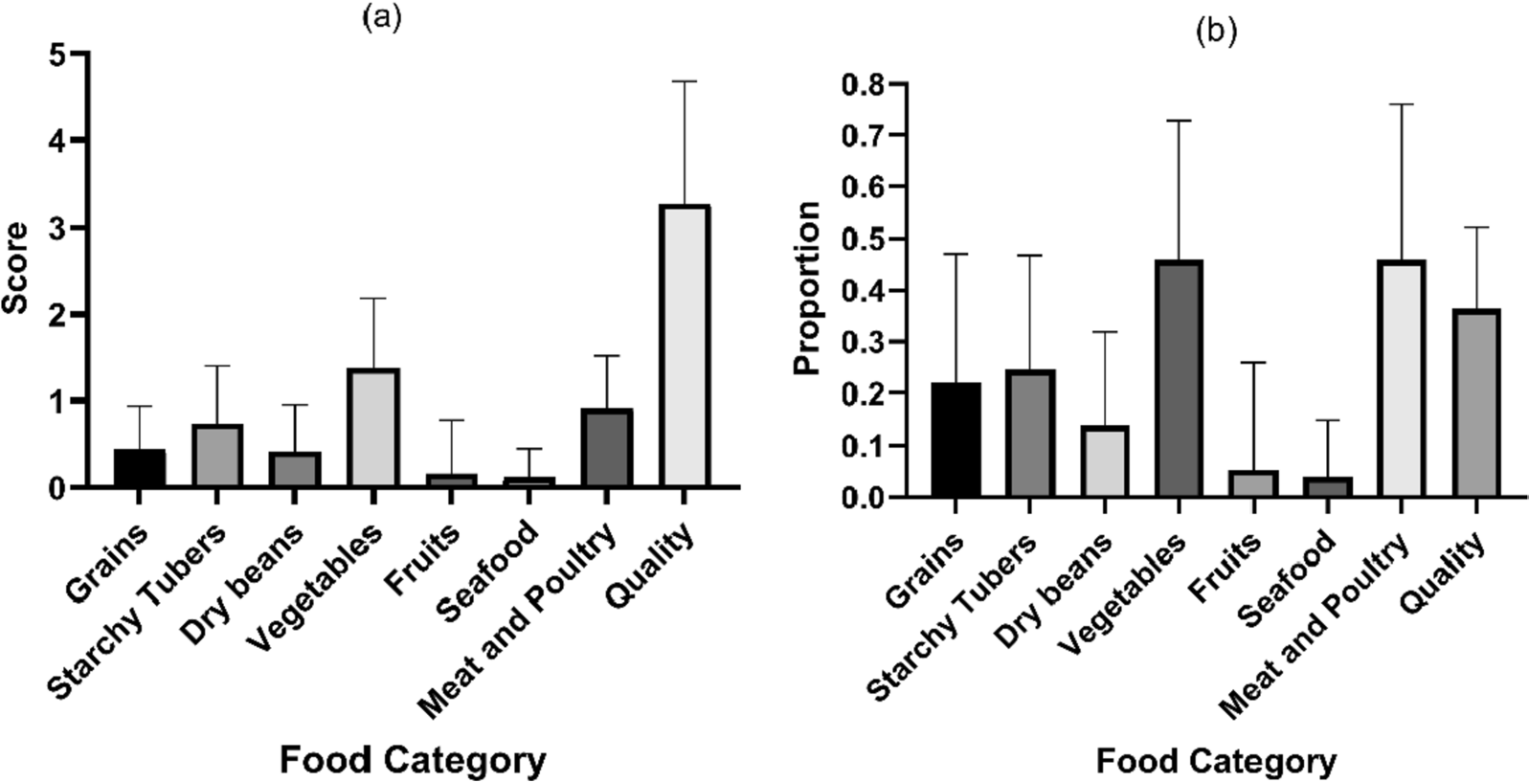
Summary histogram of C-NEMS-S scores in each food availability category. **a** Average C-NEMS-S food category availability score, it presents the original scores on the C-NEMS-S assessment; **b** Normalized C-NEMS-S food category score graph, the scores in each food category were normalized to a percentage of the maximum available score in their corresponding categories. Error bars represented the standard error of the food availability scores in each category

Then, the differences in overall food availability scores were analyzed. In Fig. 4(a), we noticed that the scores in each food category were hindered by high scores for their quality. Hence, we divided the actual scores of each food category by the maximum available scores in the corresponding category. The normalized scores were presented in Fig. 4(b). By normalizing the scores, we mitigated the issue of quality score being excessively influential within each outlet. Subsequent food availability analysis was based on the normalized scores.

**Fig. 5:**
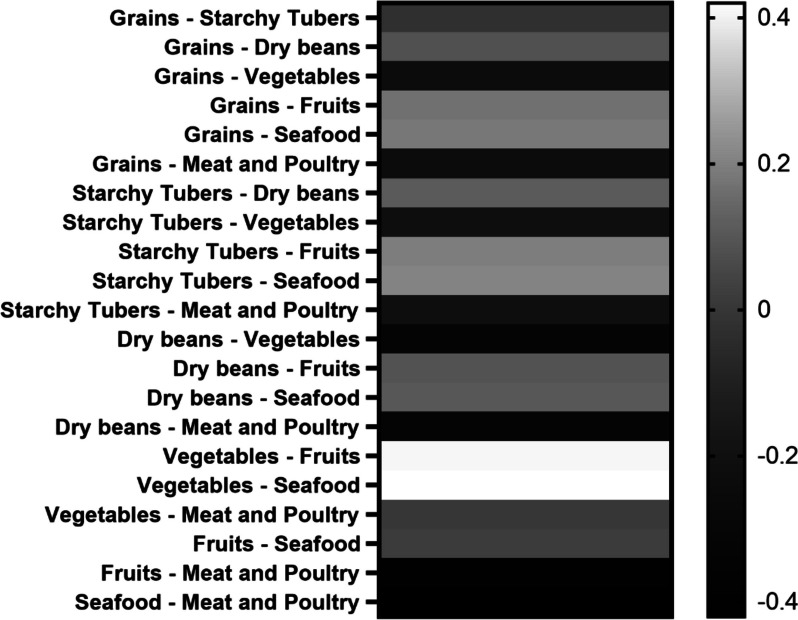
Heat map of means of normalized food availability scores. The values on the double gradient axis indicates the positive and negative mean differences in the scores shown as white and black, respectively

A one-way ANOVA analysis showed that the differences between most pairs of the food availability scores were statistically significant. All pairwise comparisons among grains, starchy tubers, and dry beans were null. The pairwise comparisons for vegetables vs. meat & poultry and fruits vs. seafood also resulted in statistically non-significant results. The heat map illustrated in Fig. 5 revealed that vegetables and meat & poultry availability scores were significantly higher than fruits, seafoods, and dry beans (all *P* < 0.0001). The author consulted the university canteen manager during an undocumented interview regarding this observation. The manager stated that seafoods and fruits had short shelf life and were perishable. Food safety remains the top priority for Chinese university canteens and seafoods and fruits were thus restricted for the outlets.

Fruits were sold in only 5 out of 52 outlets. However, a special arrangement from one of the university canteens should be noted. Canteen A offered fruits for free during lunch and dinner generally on Tuesday each week. What day the free fruit stand was present and which fruits were offered would depend on the canteen manager liaising with grocery providers, which was beyond the scope of this study. Although placed near outlet C9, the free fruit stand was not displayed on the menu, thus was part of the overall university food environment rather than of an individual outlet. For this reason, the free fruit stand was excluded from scoring but worth highlighting as an outcome of the audit.

### Healthier options & their prices

Outlets selling healthier options were scarce and appeared in “grains” (*n* 2) and “meat and poultry” (*n* 2) categories, as shown in Table 5. Price comparison was not applicable when the dishes containing regular and healthier options were substantially different in other ingredients. Fresh corn kernels were served at S07 was served as an option for spicy hot pot. The creperie pancake served at C12 was made from whole wheat and maize flour but served at a higher price than regular creperie pancakes. Lean beef was served at both C04 and C30. At C04, a direct comparison between beef and pork was observed where both meats were served with stewed potato. The prices of beef dishes were higher than equivalent pork or chicken dishes. All foods served at C30 were Halal where beef was the only meat served. Hence, the price comparison between healthier and regular options were not applicable at this outlet.

**Table 5:** Details of the four outlets that served healthier options and the price comparisons of healthier versus regular food options. CNY = Chinese Yuan

Outlet No.	Outlet Type	Healthier Option	Price (Healthier vs. Regular)
S07	Spicy Hot Pot	whole grains	Not applicable
C12	Creperie	whole grains	Higher, CNY 5 vs. 3.5
C04	Rice & Hot Dishes	lean beef	Higher, CNY 8 vs. 5
C30	Rice & Hot Dishes	lean beef	Not applicable

### Inter-rater reliability

The food environment audit results of three investigators were summarized in Table 6. One-way ANOVA suggested that the differences in mean scores were not statistically significant (*P* = 0.685). Fleiss’s kappa was calculated and the value was 0.317.

**Table 6:** Median and mean C-NEMS-S total scores from three investigators

Investigator No.	Median	Mean	95% Confidence Interval
Investigator 1	7.0	7.66	6.83, 8.49
Investigator 2	7.5	8.16	7.29, 9.03
Investigator 3	7.0	7.74	6.91, 8.57

## Discussion

### Foodservice outlet opening hours

This study offers insights into the food environment of Chinese tertiary education, particularly the opening hours of foodservice outlets, compared to Western universities [33, 34]. Chinese university canteens, constrained by tightly packed course schedules, limit food access outside mealtimes, a stark contrast to the flexibility in Western universities [35]. This difference highlights the significant role university food environments play in shaping student dietary behaviors [36].

### Food availability

Our audit reveals distinct variations in the availability of starchy tubers, dry beans, vegetables, and fruits across different campus outlet subtypes. This suggests that food choices in university canteens are influenced by the preferences of the outlets, which might compete with delivery services for student dietary purchases [37]. Traditional Chinese-style cooking typically does not include fruits as a major ingredient, reflecting the limited availability of fresh fruits in canteens due to short shelf-lives [38, 39]. Vegetables scored higher in availability, underscoring their popularity and perceived healthfulness among students and staff [40]. However, seafood availability was notably low, primarily due to the university management’s food safety concerns [41].

### Healthier options

In terms of healthier options, Chinese university food environments primarily offer whole grains and lean meats like beef, differing from Western universities that often have skim milk, diet soft drinks, fruit juice, and non-fried vegetables [22, 42, 43]. These differences are influenced by China’s grain-based dietary culture and limited beverage options in university canteens [26]. Socioeconomic factors, including income, and customer nutrition knowledge also play a role in food choices [44–46]. The university’s cost control policy, which sets a maximum profit threshold for food outlets, and the higher cost of healthier food options like grains and beef, limit the availability of diverse, healthy choices [47–50].

### Other features

The C-NEMS-S criteria provided a comprehensive overview of the food environment but missed some features like spatial arrangement, promotions, and cooking oil use, which were observed during the audit. The study found that the university canteens employed various promotional strategies and predominantly used soybean oil, impacting both dietary choices and health risks associated with high oil consumption [51–57].

### Strengths and limitations

The most prominent strength of this study is that it is, to our knowledge, the first study to use a validated audit tool to assess the food environment in a Chinese university setting. In this emerging field of food environment research, this study added the momentum to relevant research in China, especially in a university setting. Furthermore, three raters independently scored the outlets in our study. The inter-rater reliability analysis facilitated the production of reliable data, which is particularly important for pioneer research.

While the study provides valuable insights into the university food environment, certain limitations for both C-NEMS-S and the design of this study should be considered. Firstly, this study is conducted in three canteens located at one Chinese university campus only. Outcomes such as food outlet classification, C-NEMS-S criteria adjustments, and outlet score comparison methods might not be generalizable to a different university food environment in China or in another country. Secondly, the fact that C-NEMS-S is lucid and straightforward to use meant that certain information regarding the food outlets being assessed could have been neglected. Examples of neglected information included portion size, promotion, and spatial arrangements. Thirdly, C-NEMS-S offers limited details on some of the criteria included in this assessment tool, which could lead to significant bias. For instance, it does not specify the minimum amount of each food in each category to be scored on. C-NEMS-S neither assesses how significant the proportion of a food is compared to other foods in an outlet. Lastly, C-NEMS-S does not consider the cooking methods, such as water-boiled versus deep-fried, when scoring for similar food contents.

The inter-rater reliability was evaluated by Fleiss’s kappa value of 0.317, which would be categorized as “fair agreement.” However, Fleiss’s kappa has been reported to return inconsistent results by giving low kappa values when high agreement was found [58]. Although improved Fleiss’s kappa statistics and other alternative multi-rater agreement statistics have been proposed [59], they are beyond the scope of this particular audit. Nonetheless, the three markers’ understanding towards the criteria and description of C-NEMS-S could have considerably disagreed. This meant that C-NEMS-S still needs further adaptation to the Chinese university food environment to deliver more reliable outcomes. During the course of this study, the authors became aware of a new university food environment audit tool, the Nutrition Environment Scoring for Chinese Style University/Work-site Canteens (NESC-CC), developed by Han et al. [57]. This tool was not only specifically developed for auditing university food environments in China but also addressed issues like cooking methods and portion sizes, which were overlooked by C-NEMS-S.

### Implications of the findings for public health and policy

Improving palatability is crucial to the dissemination of healthier food and beverage choices [60]. The trade-off of palatability versus nutrition value or healthiness has always been going on for consumers. Meanwhile, chefs must trade cost-effectiveness for palatability where premium, tasty ingredients are often more expensive. In China, universities and their canteens usually receive government funding. Hence, food policies could provide further support for healthier options by increasing the budget specifically for such foods. Since cost-efficiency is one of the priorities that university students consider when they make dietary choices [36], supportive food policies could promote healthier eating behaviours among university students.

By increasing the variety of healthier options, students may be more likely to choose healthier foods [61]. The study found that some food outlets used less healthy cooking methods, such as frying. Food outlets could use healthier cooking methods, such as steaming or baking, to improve the overall nutritional quality of the foods offered [62, 63]. Students may be more likely to choose healthier options over less healthy ones if healthier options are offered at a lower price point [64]. Providing education and awareness campaigns on the importance of healthy eating and the benefits of consuming a balanced diet could also help to encourage students to choose healthier options [65–67].

In future research, it is crucial to expand the scope of studies on the university food environment in Chinese tertiary education settings. This study represents one of the initial investigations conducted in a large urban university in China. One avenue for future studies involves refining study design and audit tools. For instance, the NESC-CC tool assessed the food environment components in Chinese universities [57]. However, it is important to validate the improved audit tool in universities with diverse geographic regions, student demographics, and canteen cooking styles. Another direction for future research is to explore the qualitative and quantitative relationship between the university food environment and student purchase and dietary behaviors. Our research team has recently completed a study employing a diet ethnography approach to explore the decision-making processes of university students within canteen environments. Collaborative studies employing diverse approaches can contribute to a comprehensive understanding of dietary behaviors among university students, facilitating the development of effective interventions for healthier dietary choices.

## Conclusions

The C-NEMS-S tool gave insights into food availability and healthier options during the university campus food environment. It highlighted eight types of food outlets located in a Chinese university as well as assessing the food availability in these outlets. However, it needs to be extensively accustomed to the Chinese university food environments before being generalizable to other universities in China. For example, additional assessment categories could be introduced into C-NEMS-S, such as cooking methods, portion sizes, and promotion. Further research in refining current assessment methods would allow more accurate audits of university food environment. When university students are exposed to abundant dietary options, optimization of their food environment might become crucial for shaping healthy eating behaviours.

### Supplementary Information

**Additional file 1.**

## Data Availability

The dataset(s) supporting the conclusions of this article is (are) included within the article (and its Additional file(s)).
